# Extracellular vesicles from *Paracoccidioides* pathogenic species transport polysaccharide and expose ligands for DC-SIGN receptors

**DOI:** 10.1038/srep14213

**Published:** 2015-09-21

**Authors:** Roberta Peres da Silva, Christian Heiss, Ian Black, Parastoo Azadi, Jared Q. Gerlach, Luiz R. Travassos, Lokesh Joshi, Michelle Kilcoyne, Rosana Puccia

**Affiliations:** 1 Escola Paulista de Medicina-Universidade Federal de São Paulo, São Paulo, Brazil; 2 Analytical Services & Training Laboratory Complex Carbohydrate Research Center, The University of Georgia; 3 Glycoscience Group, National Centre for Biomedical Engineering Science and; 4 Microbiology, School of Natural Sciences, National University of Ireland, Galway, Ireland

## Abstract

Extracellular vesicles (EVs) mediate non-conventional transport of molecules across the fungal cell wall. We aimed at describing the carbohydrate composition and surface carbohydrate epitopes of EVs isolated from the pathogenic fungi *Paracoccidioides brasiliensis* and *P. lutzii* using standard procedures. Total EV carbohydrates were ethanol-precipitated from preparations depleted of lipids and proteins, then analyzed by chemical degradation, gas chromatography-mass spectrometry, nuclear magnetic resonance and size-exclusion chromatography. EV glycosyl residues of Glc, Man, and Gal comprised most probably two major components: a high molecular mass 4,6-α-glucan and a galactofuranosylmannan, possibly an oligomer, bearing a 2-α-Man*p* main chain linked to β-Gal*f* (1,3) and α-Man*p* (1,6) end units. The results also suggested the presence of small amounts of a (1→6)-Man*p* polymer, (1→3)-glucan and (1→6)-glucan. Glycan microarrays allowed identification of EV surface lectin(s), while plant lectin microarray profiling revealed terminal Man and GlcNAc residues exposed at the EVs surface. Mammalian lectin microarray profiling showed that DC-SIGN receptors recognized surface carbohydrate in *Paracoccidioides* EVs. Our results suggest that oligosaccharides, cytoplasmic storage, and cell wall polysaccharides can be exported in fungal EVs, which also expose surface PAMPs and lectins. The role of these newly identified components in the interaction with the host remains to be unraveled.

Paracoccidioidomycosis (PCM) is a systemic granulomatous mycosis that is endemic in rural areas of Latin America. It is caused by the temperature-dependent dimorphic species *Paracoccidioides brasiliensis* and *P. lutzii*^1^. Environmental fungal mycelia produce conidia that, once inhaled by the host, are transformed into the yeast pathogenic phase in the lungs^2^. This morphogenesis is followed by structural changes in cell wall polysaccharides, which are essential for establishment of the disease. Infected individuals may either remain asymptomatic or develop sub-clinical PCM, which can progress as an active disease in up to 2% of the cases^3^.

The fungal cell wall is mainly composed of structural polysaccharides. Fibrillar fungal cell wall polysaccharides are generally β-(1→3) and -(1→6)-glucans cross-linked to chitin via β-(1→4) bonds. In the pathogenic species *P. brasiliensis*, *Histoplasma capsulatum, Aspergillus fumigatus*, and *Cryptococcus neoformans*, the cell wall is constituted by α-(1→3)-glucan cross-linked to chitin^4^,^5^. In *Paracoccidioides*, the polysaccharide structure shifts from linear β-(1→3)-glucan with β-(1→6)-glucan short branches in mycelia to mostly α-(1→3)-glucan in the yeast phase^6^. Polysaccharide and glycoconjugate-rich cell wall constituents are a major source of pathogen associated molecular patterns (PAMPs), which are recognized by pattern-recognition receptors (PRRs). PRRs are highly expressed in the front-line immune cells, particularly macrophages and dendritic cells^7^. Fungal cells are recognized by Toll-like receptors TLR2 and TLR4, collectins SP-A and SP-D, pentraxin-3, CR3 integrin, and C-type lectins^8^. In dimorphic *H. capsulatum*, there is a mixture of α-(1→3)-glucan and β-(1→3)/(1→6)-glucan in the yeast-phase cell wall. Since the α-(1→3)-glucan localizes more superficially, it prevents binding of β-(1→3)-glucan to C-type lectin dectin-1, thus helping fungal escape from the protective immune mechanisms^9^.

Fungal extracellular vesicles (EVs) are important double-membrane structures that mediate non-conventional molecular transport across the cell wall in pathogenic fungi^10^. Fungal EVs carry not only proteins and lipids, but also capsular glucuronoxylomannan (GXM), pigments^10^, and recently described RNA^11^. Some EV components are proven virulence factors and immunomodulatory compounds^10^.

The mechanisms of eukaryotic EV formation described so far involve exosome release, formation of secretory lysosomes, and microvesicles derived from plasma membrane^12^. In addition, a new mechanism of microvesicle formation in *Saccharomyces cerevisiae* is based on reshaping of the plasma membrane due to deep invagination that results in access of the inner membrane to outer cytoplasmatic membrane and consequent release of EVs^13^. Fungal EV preparations are composed of different populations, whose biogenesis regulation seems to relate to both conventional and non-conventional protein secretory pathways^13^,^14^. In general, however, EV formation results in sequestration of neighboring cytoplasmic molecules to the EVs, thus offering a plausible explanation for the finding of proteins that lack conventional signal peptide outside the plasma membrane barrier. Extracellular proteins that lack signal peptide are largely described in the recent literature^10^. Following the conventional route, proteins are glycosylated in the endoplasmic reticulum (ER) and Golgi apparatus. Non-conventional pathways are Golgi-ER-independent and group not only vesicular transport, but also non-vesicular pathways of plasma membrane translocation across plasma membranes and transporter-based secretion^12^.

While the subject of protein/glycoprotein export has been studied for years, export of polysaccharide is poorly understood. In plants and fungi, several glycan synthases are integral membrane proteins that use monosaccharide-UDP present in the cytosol to produce directly delivered cell wall polysaccharide, as observed for α-glucans^15^ and β-glucans^16^. Some polysaccharides, however, are found in the extracellular environment. An *A. fumigatus* galactomannan is anchored to the cell wall via glycophosphatidylinositol, but it is also secreted^17^. Since its synthesis is halted by the absence of the Golgi membrane transporter for UDP-Gal*f* (GlfB), secretion is possibly associated with the secretory pathway^18^. Capsular GXM, the *C. neoformans* most important virulence factor and immunomodulator, is abundantly released to the extracellular environment and is the only polysaccharide so far described in fungal EVs^19^. GXM exported in EVs is further incorporated to the capsule; therefore EVs have a role in capsule synthesis.

Our group has recently characterized EVs isolated from culture supernatants of the yeast pathogenic phase of *P. brasiliensis*^20^. We fortuitously detected EV antigenic terminal Gal-α-(1,3)-Gal (α-Gal) epitopes, and at least part of these terminal residues seemed to compose *O*-linked oligosaccharides that ornament vesicle proteins. In order to understand the possible roles of *Paracoccidioides* EVs, we engaged in a series of works that try to elucidate what sort of molecules *Paracoccidioides* EVs carry to the extracellular environment. We have already described the *P. brasiliensis* EV proteome^21^, lipidome^22^ and, more recently, the EV RNA content in *P. brasiliensis* and other fungal species^11^.

In the present work, we aimed at characterizing the *Paracoccidioides* EV carbohydrate content. We analyzed surface EV sugar epitopes and a total carbohydrate fraction extracted from EVs isolated from the *Paracoccidioides* Pb18, Pb3, and P01 isolates. These isolates have been chosen by the Dimorphic Fungal Consortium to represent *Paracoccidioides* phylogenetic groups in the dimorphic fungal genome project of the Broad Institute. Therefore, they gained full genome information (http://www.broadinstitute.org/annotation/genome/paracoccidioides_brasiliensis/MultiHome.html). They represent the newly recognized *P. lutzii* (Pb01) species^23^, the major *P. brasiliensis* phlylogenetic S1 group (Pb18), and cryptic *P. brasiliensis* PS2 (Pb3) phylogenetic group^24^. Pb18 and Pb01 are broadly used in the literature. Pb18 is highly virulent, while Pb3 and others from the PS2 group evoke mild PCM in mice^25^. We presently analyzed the glycosyl composition and linkages, polymer sizes, and partial carbohydrate structures of total EV carbohydrates. We also used glycomics microarrays to investigate EV surface terminal oligosaccharides, ligands for mammalian lectins, and fungal lectins. Our results showed that cytoplasmic storage and cell wall polysaccharides, besides mannose oligosaccharides, are exported by fungal EVs. In addition, we detected exposed PAMPs and lectins at the EVs surface.

## Results

In order to characterize the EV carbohydrate contents and compare the results among *Paracoccidioides* species, we used an EV isolation protocol that has been well established and documented in the literature to avoid membrane contamination. In the original papers showing EV characterization in *Cryptococcus neoformans*^19^, *P. brasiliensis*^20^, and other fungal species^26^, the authors showed that the two initial centrifugation steps are able to eliminate not only cells, but also cell debris and possible apoptotic bodies^27^,^28^. There were only traces of membrane sterol in control EV preparations from culture supernatants of heat-killed yeast fungal cells^19^,^20^,^26^. In *Paracoccidioides*, the work from Vallejo *et al.*^20^ showed that after the filtration, concentration and ultracentrifugation steps, the preparations had mainly EVs sizing 20–200 nm compatible with exosomes and microvesicles, as seen in transmission electronic microscopy images. The EV yields are usually quite low; therefore, in order to proceed with partial structural analysis of carbohydrates, we needed 10 to 15 EV preparations from each isolate to gather enough total ethanol-precipitated carbohydrate out of lipid/protein-free EVs. The studies of EV-exposed epitopes via glycomics microarrays demanded significantly less amounts of isolated EVs.

### Glycosyl composition

TMS methylglycosides from *Paracoccidioides* Pb18 and Pb01 EV carbohydrate dialyzed fractions were analyzed by GC/MS. In all samples, glucose (Glc) was the main constituent (Table 1 and Supplementary Figure S1), with smaller occurrences of mannose (Man) and galactose (Gal). Residues of *N*-acetylglucosamine (GlcNAc) were not detected, suggesting that glycoproteins bearing high mannose chains are either not present in the EV fraction analyzed here or are only present in trace amounts. In addition, we have not been able to detect protein in the EV carbohydrate-enriched fractions using the BCA protein assay (Bio-Rad, not shown), suggesting that our results reflect the presence of only polysaccharide or oligosaccharide derivatives.

### Linkage analysis

**Figure 1:**
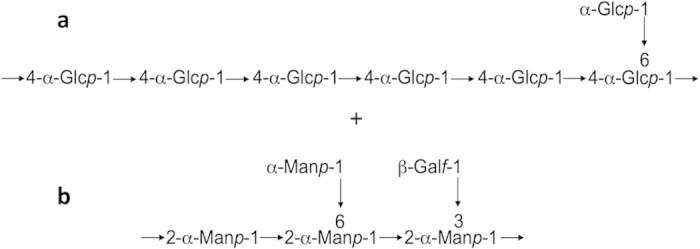
EV total carbohydrate fraction from Pb18 was analyzed for configuration of the chemical linkages by NMR (Supplementary Figure S3 and Supplementary Table S1), showing the presence of a most probable mixture of an α-(1→4)-glucan (**a**) and a galactomannanoside (**b**). The deduced structures are represented.

The PMAAS derived from total EV dialyzed carbohydrate fractions were subjected to GC-MS analysis (Table 2 and Supplementary Figure S2) and the peak areas were used to estimate the relative percentages of the residues. The most abundant peaks of the spectrum were from 4-linked glucopyranosyl residues (1,4)-Glc*p*, suggesting the presence of a (1→4)-linked Glc*p* polymer. Terminal glucopyranosyl (t-Glc*p*) was proportionally high. Minor (1,4,6)-linked Glc*p* residues suggested branching of a possible (1→4)-glucan. Indeed, further NMR analysis (see below) confirmed the presence of an α-(1→4)-glucan with α-(1→6)-linked branching (Fig. 1 and Supplementary Figure S3). In addition, the finding of 3-Glc*p* and 6-Glc*p* residues (Table 2 and Supplementary Figure S2) also suggested the presence of minor (1→3)- and (1→6)-glucans, which are structural cell wall components in *Paracoccidioides* yeasts^6^.

Terminal mannopyranosyl (t-Man*p*) and galactofuranosyl (t-Gal*f*) residues have been identified in small percentages in all samples. Significant percentages of (1,2)-Man*p* and (1,6)-Man*p* in Pb18 EVs could point to the presence of two mannose polymers.

We also analyzed Pb3 samples that have not been previously dialyzed (not shown). Therefore, we should view the results with care due to contamination with monomeric glucose from the culture medium. In general, however, we observed that glucose residues predominated also as (1,4)-Glc*p*, while (1,3)-Glc*p* and (1,2)-Man*p* indicated the presence of other polymers. High amounts of t-Glc*p*, and small percentages of t-Man*p*, and t-Gal*f* have been observed.

### NMR analysis

NMR spectroscopy was carried out with the dialyzed whole EV carbohydrate fraction from Pb18. The result revealed that the major component was 4-linked α-Glc*p*, which makes up the backbone of the polysaccharide (Supplementary Table S1). A minor amount (about one sixth of the 4-Glc) of terminal α-Glc*p* was also found. Although we could not clearly observe an NOE contact to H-6 of (4,6)-α-Glc*p* (Residue H), due to the overwhelming intra- and inter-residue NOE cross peaks of 4-Glc*p* (Residue B), linkage analysis suggested that t-Glc (Residue A) was *O*-6-linked to some residues of the polysaccharide skeleton (Supplementary Figure S3). We detected several α-Man*p* and β-Gal*f* residues at lower intensity as well. Although we have been able to identify each of these residues, assign most of their chemical shifts, and determine some of the linkages between residues through inter-residue NOEs, we could not unambiguously elucidate the complete sequence. There are two likely possibilities regarding the structure of the polysaccharide and based on the fungal literature we favored the model in Fig. 1, which consists of a mixture of two polysaccharides: 4,6-α-glucan and a galactofuranosylmannan bearing an 2-α-Man*p* main chain linked to a non reducing α-Gal*f* (1,3) end unit and an β-Man*p* (1,6) end unit. A second structure not favored by the literature would be a 4,6-α-glucan that has additional galactofuranosylmanno-oligosaccahride side chains attached to *O*-6 Glc.

Size-exclusion chromatography of the Pb18 EV carbohydrate fraction was performed after the aforementioned data aquisition and resulted in three major peaks (Supplementary Figure S4). A high molecular mass fraction of >1,200 kDa corresponded to 29.8% of the area. Peaks 2 and 3 (about 20% each) corresponded to oligomers of around 1 kDa.

Together, the methylation, size-exclusion chromatography, and NMR data pointed to the existence of a major storage 4,6-α-glucan and we could speculate that it is compatible with the high molecular mass peak. We could also speculate that the galactofuranosylmannan possibly corresponds to the more abundant oligomer peak.

### Plant lectin microarray profiling

**Figure 2:**
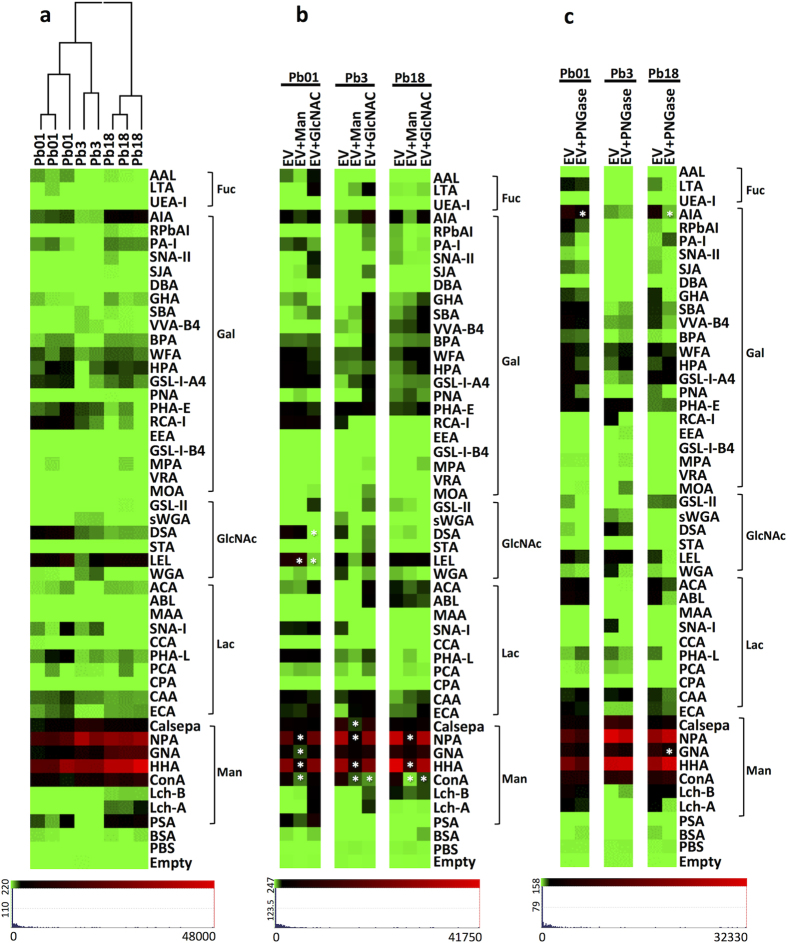
Lectin microarray for intact EVs from Pb18, Pb3, and Pb01. Data were normalized and the scale is indicated below the figures. (**a**) Unsupervised hierarchical clustering was performed for individual technical replicates. (**b**) Competitive inhibition of EV interactions with lectin microarray with mannose (Man) and *N*-acetylglucosamine (GlcNAc). (**c**) Lectin microarray of PNGase F-treated EVs. Significance (* = p ≤ 0.05) was determined by the ANOVA test.

To investigate the terminal oligosaccharides exposed in the *Paracoccidioides* EVs surface, we performed lectin microarray profiling using preparations from isolates Pb18, Pb3, and Pb01. We observed signal intensities ranging from 100 to about 40,000 fluorescence intensities (RFU) for 31 (Pb18), 29 (Pb01), and 23 (Pb3) lectins (Fig. 2a). Binding to Man-specific lectins showed higher intensity with *Paracoccidioides* EVs, but some interaction was observed for Gal-, lactose (Lac)-, fucose (Fuc)-, and GlcNAc-binding lectins (Supplementary Table S2). The profiles of lectin binding were generally similar among isolates, even though the fluorescence intensities varied considerably (Fig. 2a). Therefore, the patterns of terminal oligosaccharide on the EV surface were similar, but the concentrations expressed seemed to vary with the isolate, perhaps reflecting export of EV populations that have distinct biogenesis. Hierarchical clustering of the glycosylation profile showed that Pb01 and Pb3 are more similar to one another than Pb18. To confirm the specificity of these interactions, we performed inhibition assays with Man and GlcNAc (Fig. 2b). EV binding to Man and GlcNAc-specific lectins was inhibited with Man and GlcNAc, suggesting that *Paracoccidioides* EV surface is mainly composed by high amounts of terminal Man oligosaccharides and lower amounts of terminal GlcNAc.

*N*-linked glycans are covalently attached to protein at the asparagine residues with GlcNAc via an *N*-glycosidic bond^29^ and the intact oligosaccharides may be released by PNGase F treatment. In order to investigate if the terminal oligosaccharides detected on the EV surface with lectins were components of *N*-linked glycans, we treated Pb18, Pb3, and Pb01 EVs with PNGase F and profiled them on lectin microarrays. The results revealed a mild decrease in binding to terminal Man (GNA or Lch A), Lac (ECA or PHA-L), GlcNAc (LEL or WGA), Gal (AIA, RPbAI, GHA, WFA or PNA), and Fuc-specific lectins (LTA) after enzymatic treatment (Fig. 2c). This slight reduction in binding suggested that part of the terminal oligosaccharides are attached to *N*-linked glycans of membrane glycoproteins.

### Mammalian lectin microarray profiling

**Figure 3:**
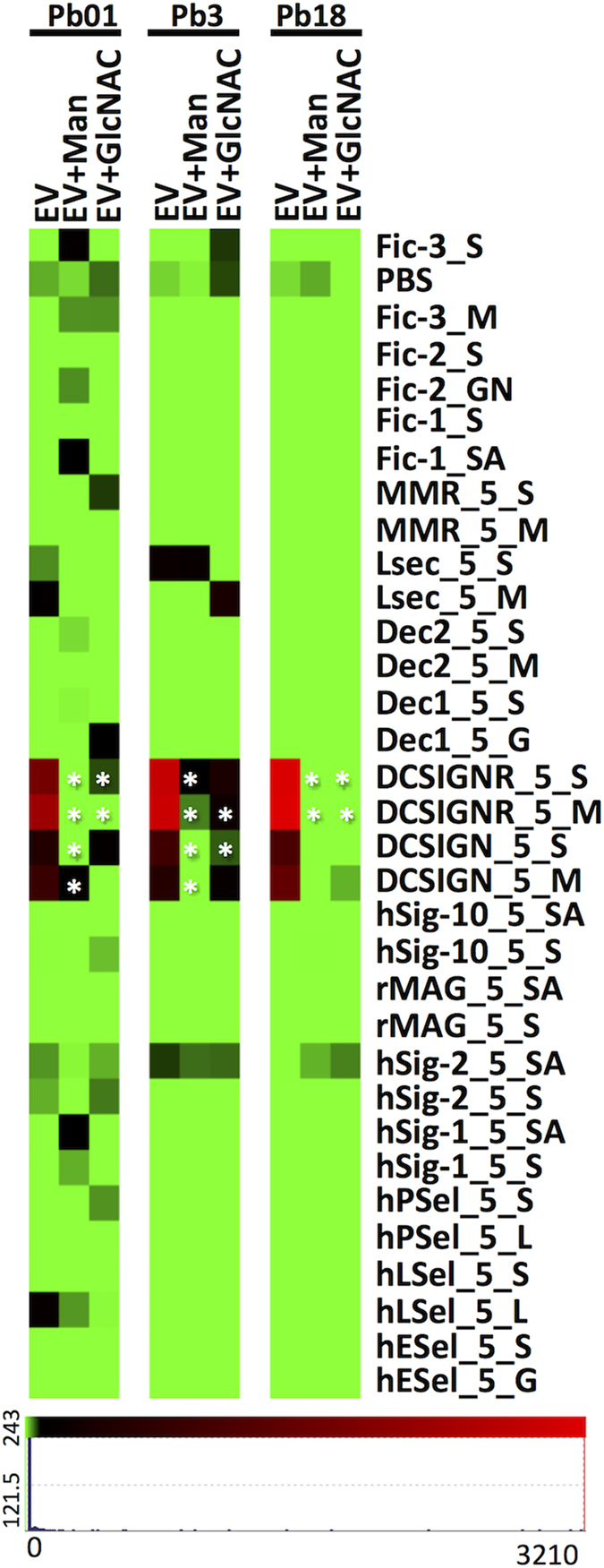
Mammalian lectin microarrays for Pb18, Pb3, and Pb01 EVs and competitive inhibition of interactions with with mannose (Man) and *N*-acetylglucosamine (GlcNAc). Averages of normalized individual technical replicates were plotted and the scale is indicated below the figure. The ANOVA test was used to verify significance (* = p ≤ 0.05).

We tested the binding of *Paracoccidioides* EVs with 10 mammalian C-type lectins (CTLs), 4 I-type lectins and 3 human Ficolin receptors. EVs from Pb18, Pb3 and Pb01 showed similar profiles of binding especially to DC-SIGN and DC-SIGNR (Fig. 3). DC-SIGN is a C-type receptor mainly expressed by myeloid dendritic cells. It typically recognizes exposed Man, GlcNAc, and Fuc-containing glycans, besides pathogen polysaccharides, which results in internalization, processing and presentation of pathogen-derived antigens^30^,^31^. DC-SIGNR is expressed on sinusoidal endothelial cells and binds to the same terminal oligosaccharides. We also observed low signals of binding to human siglec-2 (B cells), L-sectin (sinusoidal endothelial cells), and human L-selectin (lymphocytes) for Pb01. Inhibition assays were initially performed for Pb18 EV preparations in the presence of Man, Gal, Fuc, GlcNAc, Lac or cow IgG (data not shown). Decreased signals were mainly observed upon co-incubation with Man, GlcNAc, and Fuc. Inhibition assays for Pb3 and Pb01 EVs were carried out only with Man and GlcNAc, as these were the two main terminal residues found in these samples. Both prompted a decrease in fluorescence intensity (Fig. 3). These results suggested that the interaction between EV surface and DC-SIGN/DC-SIGNR is carbohydrate-mediated.

### Glycan microarray

**Figure 4:**
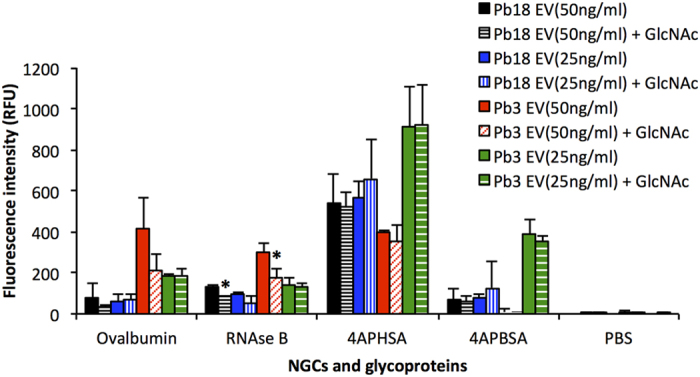
Glycan microarray. The graph shows binding profiles of Pb18 and Pb3 PKH26-labelled EVs at 50 and 25 ng/ml and neoglycoconjugates (NGCs) or glycoproteins printed in the Nexterion Slide H. The X-axis shows fluorescence intensity of EVs binding to specific NGCs or glycoproteins indicated in the Y-axis. (*), statistically significant (*p *≤ 0.05).

To detect EV surface proteins (lectins) capable of binding to carbohydrate, we carried out glycan microarray analysis for Pb18, Pb3, and Pb01 EVs. We observed low intensities of binding to 40 neoglyconjugates or glycoproteins to Pb3 and Pb01 EVs; Pb18 EVs showed only 33 interactions (data not shown). Inhibition assays were performed with 50 mM Man, Gal, Fuc, Lac, xylose (Xyl) (data not shown), or GlcNAc (Fig. 4). There was binding inhibition only with GlcNAc, thus confirming the nature of a carbohydrate-mediated interaction, however only at the highest EV concentration (50 ng/ml). Interactions with Pb01 EVs were performed only at 25 ng/ml and they were not inhibited by any monosaccharide. These results pointed to the existence of EV surface lectin(s) recognizing GlcNAc.

## Discussion

We presently found polysaccharides, possible oligosaccharides, and surface terminal oligosaccharide residues in extracellular vesicles (EVs) released by the pathogenic yeast phase of *Paracoccidioides* representative species. EV glycosyl residues of Glc, Man, and Gal comprised most probably two major components: a high molecular mass 4,6-α-glucan and a galactofuranosylmannan polymer, possibly an oligomer, bearing a 2-α-Man*p* main chain linked to β-Gal*f* (1,3) and α-Man*p* (1,6) end units. The results also suggested the presence of small amounts of a (1→6)-Man*p* polymer and characteristic cell wall (1→3)-glucan and (1→6)-glucan. Glycan microarrays revealed EV surface GlcNAc-binding lectin(s), while plant lectin microarray profiling revealed terminal Man and GlcNAc residues exposed at the EVs surface. Mammalian lectin microarray profiling showed that DC-SIGN receptors recognized surface carbohydrate in *Paracoccidioides* EVs.

Linear α-(1→4)-glucans, branched or not with α-(1→6)-Glc residues, are typical cytosolic storage polysaccharides. Poorly branched or linear α-(1→4)-glucans are characteristic of plant starch (amylose and branched amylopectin), while densely branched glycogen is observed in most eukaryotic and prokaryotic organisms^32^. We have identified by NMR a possible glycogen fragment composed of α-(1→4)-glucan with a single α-(1,6)-Glc*p* side chain in *Paracoccidioides* EVs. We envision that either glycogen^33^ and/or its hydrolysis products are stolen from the cytoplasm in the process of EV formation and transported inside the vesicles to the cell wall and extracellular environment. Although we have not analyzed the size-exclusion peaks from EVs carbohydrate, glycogen is likely to correspond to the high molecular mass polysaccharide, considering that masses over 1,200 kDa are characteristic of storage carbohydrate.

In *Saccharomyces*, glycogen can be linked to β-glucan in the cell wall fibrillar structure^33^. On the other hand, α-(1→4)-Glc*p* interconnecting residues can be found in the structural cell wall α-(1→3)-glucan from *Aspergillus*, *Fusicoccum amygdali*, *Neurospora crassa*, and *C. neoformans*^34^,^35^,^36^,^37^. In yeast phase *Paracoccidioides* cell wall, there are single side α-(1→4)-Glc*p* residues linked to the main α-(1→3)-glucan^38^. In *H. capsulatum,* and also in *P. brasiliensis*^39^, an α-1,4-amylase (Amy1p) is essential for fungal cell wall growth and host infectivity^40^. In both species, the Amy1p seems to mediate hydrolysis of α-(1→4)-glucan polysaccharides. *P. brasiliensis* Amy1p produces malto-oligosaccharides that could be involved in the initiation of α-(1→3)-glucan synthesis or in the process of transglycosylation^40^,^41^. Despite the fact that Amy1p has not been detected in the secretome of yeast-phase *P. brasiliensis*^21^ or *H. capsulatum*^42^, enzymes responsible for carbohydrate metabolism, such as cell wall α-1,3-glucan synthase mok13 (PADG_03169), endo-β-1,3-glucanase (PADG_07351), glucan 1–3-β-glucosidase (PADG_07615), and α-mannosidase (PADG_04148) are transported by EVs^21^, suggesting that EV polysaccharides and carbohydrate metabolism enzymes could have role in glucan synthesis and remodeling of the cell wall, as well as in lipid and protein glycosylation. So far, GXM has been the only polysaccharide described in EVs and its role in *C. neoformans* capsule synthesis has been demonstrated^19^. We presently showed the presence of intracellular and cell wall polysaccharides in fungal EVs and speculate about their role in cell wall synthesis, assuming that some EVs might remain in this compartment on the way outwards. Our results suggest the inclusion of *P. brasiliensis* in a very limited group of organisms that could supposedly synthesize polysaccharide intracellularly for further extracellular export^18^,^19^.

The nature of the oligomers presently identified by size exclusion chromatography in EVs is unknown. Albuquerque and colleagues^43^ identified a GXM-like molecule in a cell wall DMSO fraction from *P. brasiliensis*. This molecule could be incorporated onto the cell surface of the hypocapsular mutant and formed a capsule-like structure that conferred protection against phagocytosis and reacted with anti-GXM MAb 18B7. The antibodies also reacted with *P. brasiliensis* EV components^43^. Since MAb18B7 can recognize the galactomannan-containing conidia of *A. fumigatus,* as well as polysaccharides from *H. capsulatum*^43^ that share carbohydrate epitopes from M and H glycoproteins with galactomannan^44^, we suggest that the *Paracoccidioides* EV galactofuranosylmannan polymer is reacting with this MAb18B7. On the other hand, trace amounts of (1,4)-xylose have been detected in Pb18 and Pb01 EVs, and we should further investigate the nature of the carbohydrate chains containing xylose.

The present glycomics microarray analysis showed that there are Man and GlcNAc residues at the *Paracoccidioides* EV surface that seemed to be recognized by the innate immune receptors DC-SIGN and DC-SIGNR, but not dectin-1 or -2. DC-SIGNR plays a role in cell adhesion, cell signaling, primary immune response and pathogen internalization^45^. It remains to be tested if *Paracoccidioides* EVs have a role in pathogen-host communication with the innate immune system. Indeed, EVs from *Candida albicans* are internalized by macrophages and dendritic cells, where they stimulate cytokine production and upregulate the MHC-II and CD86 expression^46^.

Surprisingly, we could not detect any accessible terminal α-linked Gal on the *Paracoccididoides* EV surface with MOA by lectin microarrays. EVs have not bound to the EEA, GS-I-B4, MPA and VRA lectins either, although they are also specific for terminal α-linked Gal (in the case of GS-I-B4 it is exclusive and finely specific for this motif), thus confirming the lack of accessible terminal α-linked Gal on the EV surface. Previous *Paracoccidioides* TEM images^20^ of EVs labeled with anti-α-Gal suggested intravesicular and also surface localization of α-Gal epitopes. By revisiting those results, we observed that we used 5-nm MOA gold particles. Considering that plasma membranes are 3–5 nm wide^47^, tagged α-Gal epitopes that are within the membrane or near the membrane would visually seem to be on the surface, even if not exposed. That would be a plausible explanation for the apparently contradictory results.

We presently detected lectin(s) at the *Paracoccidioides* EV surface that could lead to another type of interaction with the host. Since binding was inhibited by GlcNAc, this lectin could be the 70 kDa paracoccin, a GlcNAc-binding lectin exported to the *P. brasiliensis* yeast cell wall and to culture supernatants^48^,^49^. Paracoccin binds to laminin and induces strong and persistent production of TNF-α and nitric oxide by macrophages^48^. Mice treated with paracoccin have decreased pulmonary fungal burden and granulomas, both associated with lower levels of IL-12 and IFN-γ^50^. In addition, paracoccin is involved in chitin distribution, cell wall organization^49^, and morphogenesis^51^. Paracoccin was identified as a hypothetical protein (PADG-3347) that has enzymatic and lectin domains of endochitinases family 18^50^, but we could not detect its sequence in the *P. brasiliensis* EV proteome^21^.

We believe that the present work brings relevant contribution to the literature related to unconventional molecular export using EVs, considering we showed that: a) high levels of storage fungal polysaccharide originally located in the cytoplasm can apparently be exported across the cell wall within EVs; b) possible Man oligomers and fungal cell wall structural polysaccharides can also be exported in EVs; c) carbohydrate PAMPs are exposed at the EV surface; d) fungal lectins can also be at the EV surface. Future work will help understand the biological functions of the EV carbohydrate contents and exposed epitopes.

## Materials and Methods

### Fungal strains and culture conditions

*P. brasiliensis* isolates Pb18, Pb3, and *P. lutzii* Pb01 were maintained in the yeast phase at 36 °C in slants of modified YPD medium (0.5% yeast extract, 0.5% casein peptone, 1.5% glucose, pH 6.5). For isolation of EVs, yeast cells from 7-day-old slants were transferred to Erlenmeyer flasks containing 200 mL of Ham’s F12 medium (Invitrogen), supplemented with 1.5% glucose (Sigma), and cultivated for 4 days at 36 °C with shaking. Yeast cells from four flasks were allowed to sediment at the bottom by gravity, supernatant fluids were carefully removed, the cells were transferred to flasks containing 500 mL of fresh medium and cultivated for another 48 h.

### Extracellular vesicle (EV) isolation

EVs were isolated according to Vallejo *et al.*^20^. Briefly, *Paracoccidioides* yeast cells were separated from supernatant fluids following two centrifugations at 4 °C (4,000 *g* for 15 min and 15,000 *g* for 30 min). The resulting supernatants were 20-fold concentrated, centrifuged at 15,000 *g* for 30 min, and ultracentrifuged at 100,000 *g* for 1 h to pellet vesicles. EV sterol and protein contents were estimated using the Amplex Red Cholesterol kit (Invitrogen) and BCA Protein Assay Kit (Pierce), respectively. EV preparations were stored at −20 °C until use.

### Carbohydrate extraction from EVs

EV preparations were concentrated by centrifugal evaporation under reduced pressure (SpeedVac, Eppendorf). Total lipids were removed following three extractions with chloroform:methanol (2:1, v-v) and three with chloroform:methanol (9:1, v-v). The resulting pellets were resuspended in 0.05% trifluoroacetic acid (TFA) and loaded onto a zip-tip column containing 2 mg of POROS 50 R2 (Applied Biosystems) activated with methanol and equilibrated with 0.05% TFA. Total carbohydrates were recovered from the flow-through by adding five volumes of ethanol, followed by overnight incubation on ice and centrifugation at 18,000 *g* for 30 min. The precipitate was suspended in water and stored at −20 °C. The carbohydrate content was estimated by the phenol-sulfuric method using glucose (Glc) as standard^52^.

### Glycosyl composition

Glycosyl composition of the polysaccharide fraction obtained from EVs was performed by combined gas chromatography/mass spectrometry (GC/MS) of the per-*O*-trimethylsilyl (TMS) derivatives of the monosaccharide methyl glycosides resulting from acidic methanolysis, as previously described^53^. Total sample was suspended in deionized water and placed in 1-kDa MWCO dialysis bag (Spectrapor, RC) and dialyzed under running de-ionized water for 48 h. Dialyzed samples free of lower MW fractions were repeatedly freeze-dried for further analysis.

Briefly, methyl glycosides were prepared from dry samples (400 μg) by methanolysis in 1 M HCl in methanol at 80 °C for 18 h, followed by detection of amino sugars by re-*N-*acetylation with pyridine and acetic anhydride in methanol. The samples were then per-*O*-trimethylsilylated by treatment with Tri-Sil (Pierce) at 80 °C for 30 min. GC/MS analysis of the TMS methyl glycosides was performed in an Agilent 6890N GC interfaced to a 5975B MSD, using an Agilent DB-1 fused silica capillary column (30 m × 0.25 mm ID).

### Linkage analysis

For glycosyl linkage analysis, EV polysaccharide fractions (500 μg - 1 mg) were suspended in 200–300 μl of dimethyl sulfoxide (DMSO) and left to stir for one week. The samples were then permethylated upon addition of sodium hydroxide and methyl iodide in dry DMSO, as described^54^. The permethylated samples were hydrolyzed in 2 M TFA, reduced with sodium borohydride, and acetylated using acetic anhydride/TFA, thus producing partially methylated alditol acetates (PMAAs). The resulting PMAAS were analyzed on a Hewlett Packard 5975C GC interfaced to a 7890A MSD (mass selective detector, electron impact ionization mode); separation was performed on a 30 m Supelco 2330 bonded phase fused silica capillary column.

### Nuclear Magnetic Resonance (NMR) Spectroscopy

The NMR analysis was performed only with the Pb18 whole EV carbohydrate fraction. The deuterium-exchange was achieved by dissolving 800 μg of sample in 270 μl deuterium oxide (D_2_O), lyophilisation to dryness and immediate suspension in 99.99% D_2_O. One dimensional (1-D) ^1^H and two dimensional (2-D) gCOSY, TOCSY, NOESY, and gHSQC NMR spectra were obtained on a Varian Inova-600 MHz spectrometer at 60 °C using standard Varian pulse sequences. TOCSY and NOESY mixing times were 80 and 400 ms, respectively. Proton chemical shifts were measured relative to internal acetone (δ_H _= 2.218 ppm, δ_C _= 33.0 ppm). Preliminary results revealed a significant amount of monomeric Glc interfering in the NMR analysis. Interference was eliminated after dialysis against water using a 1.0 kDa membrane.

### Size-exclusion chromatography (SEC)

Pb18 EV (1 μg) was dissolved in 200 μl de-ionized water, filtered through 0.45 μm and loaded (20 μl) onto a Superose 6 PC 30/100 GL column (GE-Amersham) equilibrated with 50 mM ammonium acetate pH 5.5 buffer, using Agilent Technologies 1200 LC system. Seven types of standard dextran with molecular weights of 1,200, 1,135, 759, 511, 10, 5, 1 kDa and inositol (Vi) were used to calibrate the column. Data collection and processing was performed by Agilent ChemStation software.

### EV fluorescent labeling

EV preparations were labeled with PKH26 red fluorescent membrane linker-dye (Sigma) as previously described^55^ and according to the manufacturer’s protocol with minor modifications. In brief, purified EVs (200 μL) corresponding to 100 μg of protein were labeled by adding 800 μL diluent C (Sigma linker kit) and 1 ml diluted PKH26 dye. After 5 min of incubation, reactions were interrupted with 2 mL of 1% BSA. Labeled EVs were washed in PBS, pelleted by ultracentrifugation at 100,000 *g* for 1 h, suspended in a final volume of 400 μL and stored at −20 °C. EV membranes were fluorescently labeled with PKH26 and EV binding to the immobilized lectins were recorded as fluorescence intensity.

### Construction of glycomics microarrays

A panel of forty eight plant, fungal and animal lectins were selected for inclusion in the lectin microarray based on their reported carbohydrate binding specificities (Supplementary Table S2) and for glycan microarrays, a panel of 74 neoglyconjugates and glycoproteins comprising a variety of monosaccharides and oligosaccharides relevant to mammalian glycosylation were selected for printing (Supplementary Table S3). For mammalian lectin (or pathogen recognition receptor) microarrays, a panel of 16 recombinant and Ig-fusion mammalian lectins were reconstituted in phosphate buffered saline, pH 7.4 with 0.05% periodate-treated^56^ human serum albumin (HSA) (Supplementary Table S4). Probes (neoglyconjugates, glycoproteins and lectins) were printed on Nexterion® Slide H (Schott, Mainz, Germany) using a SciFlexArrayer S3 piezoelectric microarray printer (Scienion, Berlin, Germany) under constant 62% (+/−2%) humidity at 20 °C essentially as previously described^57^,^58^,^59^. For all microarrays, six replicate features per probe were printed, with approximately 1 nL per feature, using an uncoated 90 μm glass dispenser capillary and eight replicate subarrays were printed per microarray slide. After printing, slides were incubated in a humidity chamber overnight to ensure complete conjugation. The slides were blocked by incubation in 100 mM ethanolamine in 50 mM sodium borate, pH 8.0 for 1 h, then washed three times in PBS, pH 7.4, with 0.05% Tween-20 (PBS-T) for 5 min each wash and once with Tris-buffered saline supplemented with Ca^2+^ and Mg^2+^ ions (TBS; 20 mM Tris-HCl, 100 mM NaCl, 1 mM CaCl_2_, 1 mM MgCl_2_, pH 7.2). Slides were dried by centrifugation at 1,500 rpm for 5 min and stored at 4 ^o^C with desiccant until use.

### Microarray incubations

All microarray slides were incubated using an 8 well gasket slide and incubation cassette system (Agilent Technologies) and were protected from light throughout the procedure. Incubations with labeled EVs were carried out essentially as previously described^55^,^57^,^58^. In brief, labeled EVs (70 μL in TBS with 0.05% Tween-20 (TBS-T)) were loaded on to each well of the gasket slide at a concentration range of 0.75 μg/mL for lectin microarrays, 50 or 25 ng/mL for glycan microarrays and 250 ng/mL for mammalian lectin microarrays based on EV protein concentration. To verify carbohydrate-mediated binding^60^, incubations were performed in parallel in presence of 50 mM GlcNAc or methyl α-D-mannopyranoside in TBS-T for mammalian lectin microarrays, 50 mM Man, Gal, Fuc, Lac, Xyl or GlcNAc for glycan microarrays and 50 mM Man, Gal, Fuc, GlcNAc, Lac or 5 mg/mL of bovine IgG for lectin microarrays to test for binding inhibition. Fetuin was used as control. To detect the participation of *N*-linked glycans in binding to the lectin microarrays, 750 ng of labeled EVs were pre-treated with 500 U of PNGase F for 1 h at 37 ^o^C. After loading the gaskets, the microarray slides were sandwiched with the gasket, the cassette was assembled and placed in a rotating incubation oven at approximately 4 rpm at 23 ^o^C for glycan and lectin microarrays or 37 ^o^C for mammalian lectin for 1 h. Three replicate slides were incubated per experiment. Slides were disassembled in TBS-T, washed three times in TBS-T for 2 min each with gentle agitation in a Coplin jar and then once in TBS. The microarrays were dried by centrifugation (1,500 rpm, 5 min) and scanned immediately in an Agilent microarray scanner.

### Microarray data extraction and analysis

Raw intensity values were extracted from the image .tif files using GenePix Pro v6.1.0.4 (Molecular Devices, Berkshire, U.K.) essentially as previously described^57^,^58^,^59^. Results were analyzed in Excel (v.2011, Microsoft) where normalization and all data calculations were performed as described^57^,^58^. Binding data is represented in histogram form of average intensity with one standard deviation (SD) of six replicates from three experiments (n = 18). Unsupervised clustering of normalized binding data was performed with Hierarchical Clustering Exploer v.3.5 (Human–Computer Interaction Lab, University of Maryland, http://www.cs.umd.edu/hcil/hce/hce3.html) as previously described^57^. Statistical Analysis (a one-way ANOVA) was performed to evaluate significant decrease in EV binding in presence of monosaccharide tested.

## Additional Information

**How to cite this article**: Peres da Silva, R. *et al.* Extracellular vesicles from *Paracoccidioides* pathogenic species transport polysaccharide and expose ligands for DC-SIGN receptors. *Sci. Rep.* **5**, 14213; doi: 10.1038/srep14213 (2015).

## Supplementary Material

Supplementary Information

## Figures and Tables

**Table 1:** GC-MS glycosyl composition of total EV carbohydrates from Pb18 and Pb01 yeast cells after derivatization into TMS methylglycosides.

Glycosyl residue	**Mol %**^1^	
	**Pb18**	**Pb01**
Man	21.6	2.4
Gal	2.4	−
Glc	76.0	97.6

The table shows total percentages of Man, Gal, and Glc identified in each sample.

^1^Values are expressed as mole percent of total carbohydrate. The total percentage may not add to exactly 100 % due to rounding.

**Table 2:** GC-MS analysis of glycosyl linkages of PMAAS derived from Pb18 and Pb01 EV total carbohydrates.

**Glycosyl residue**	**Peak area (%)**	
	**Pb18**	**Pb01**
Terminal Mannopyranosyl residue (t-Man)	0.7	0.2
Terminal Glucopyranosyl residue (t-Glc)	7.4	7.2
Terminal Galactofuranosyl residue (t-Galf)	2.3	0.3
4 linked Xylopyranosyl residue (4-Xyl)	0.1	0.3
3 linked Glucopyranosyl residue (3-Glc)	0.4	0.2
2 linked Mannopyranosyl residue (2-Man)	5.2	0.6
4 linked Mannopyranosyl residue (4-Man )	0.9	2.4
6 linked Mannopyranosyl residue (6-Man )	2.3	0.3
6 linked Glucopyranosyl residue (6-Glc)	0.1	−
4 linked Glucopyranosyl residue (4-Glc)	71.2	81.9
2,3 linked Mannopyranosyl residue (2,3-Man )	1.8	0.3
3,4 linked Glucopyranosyl residue (3,4-Glc)	0.3	0.4
3,6 linked Mannopyranosyl residue (3,6-Man )	0.2	−
2,6 linked Mannopyranosyl residue (2,6-Man )	0.5	−
4,6 linked Glucopyranosyl residue (4,6-Glc)	6.6	5.8

The percentages of peak areas are shown for each linkage.
