# Supplementary material for: Extracellular vesicles from Paracoccidioides pathogenic species transport polysaccharide and expose ligands for DC-SIGN receptors
Source: Sci Rep. 2015 Sep 21;5:14213. doi: 10.1038/srep14213 (PMC4585699; doi:10.1038/srep14213)
Supplement: Supplementary Information [file srep14213-s1.pdf]

Extracellular vesicles from *Paracoccidioides* pathogenic species transport polysaccharide and expose ligands for DC-SIGN receptors

Roberta Peres da Silva[1], Christian Heiss[2], Ian Black[2], Parastoo Azadi [2], Jared Q. Gerlach[3], Luiz R. Travassos[1], Lokesh Joshi[3], Michelle Kilcoyne[3,4], Rosana Puccia[1]*

[1] Escola Paulista de Medicina-Universidade Federal de São Paulo, São Paulo, Brazil; [2]Analytical Services & Training Laboratory Complex Carbohydrate Research Center, The University of Georgia; [3]Glycoscience Group, National Centre for Biomedical Engineering Science and [4]Microbiology, School of Natural Sciences, National University of Ireland, Galway, Ireland

*Corresponding author:

E-mail address: ropuccia@gmail.com

Phone: +55 11 5576-4551 ext. 1508

Supplementary Material

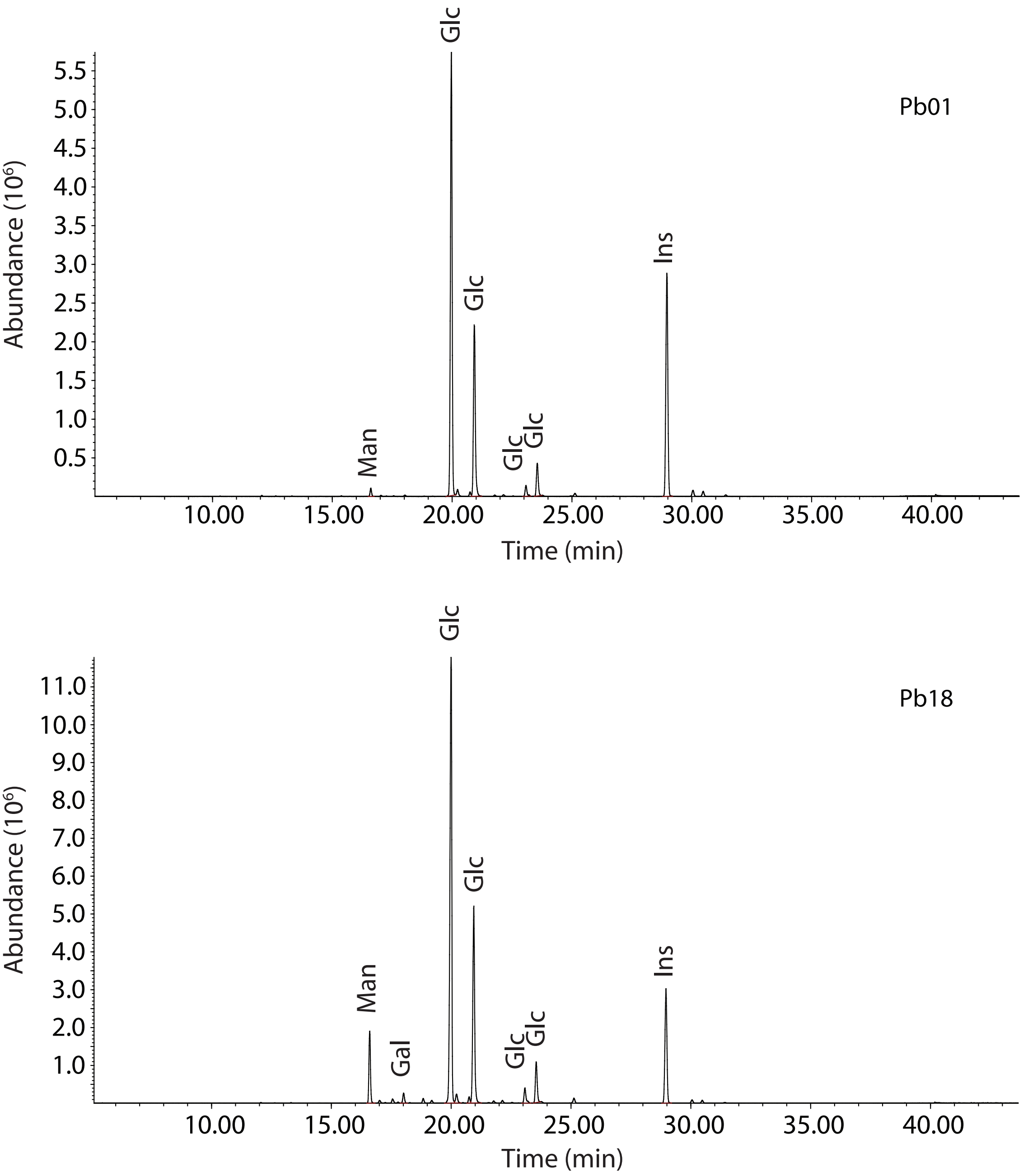

Supplementary Figure 1: GC-MS glycosyl profiles of dialyzed EV total carbohydrate fractions from Pb18 and Pb01 yeast phase after derivatization into TMS methylglycosides. The chromatograms show the relative abundance of resulting monomers (indicated) for each preparation versus the relative retention times (min) in the fused silica capillary column. Inos, inositol-internal standard; Man, mannose; Gal, galactose; and Glc, glucose.

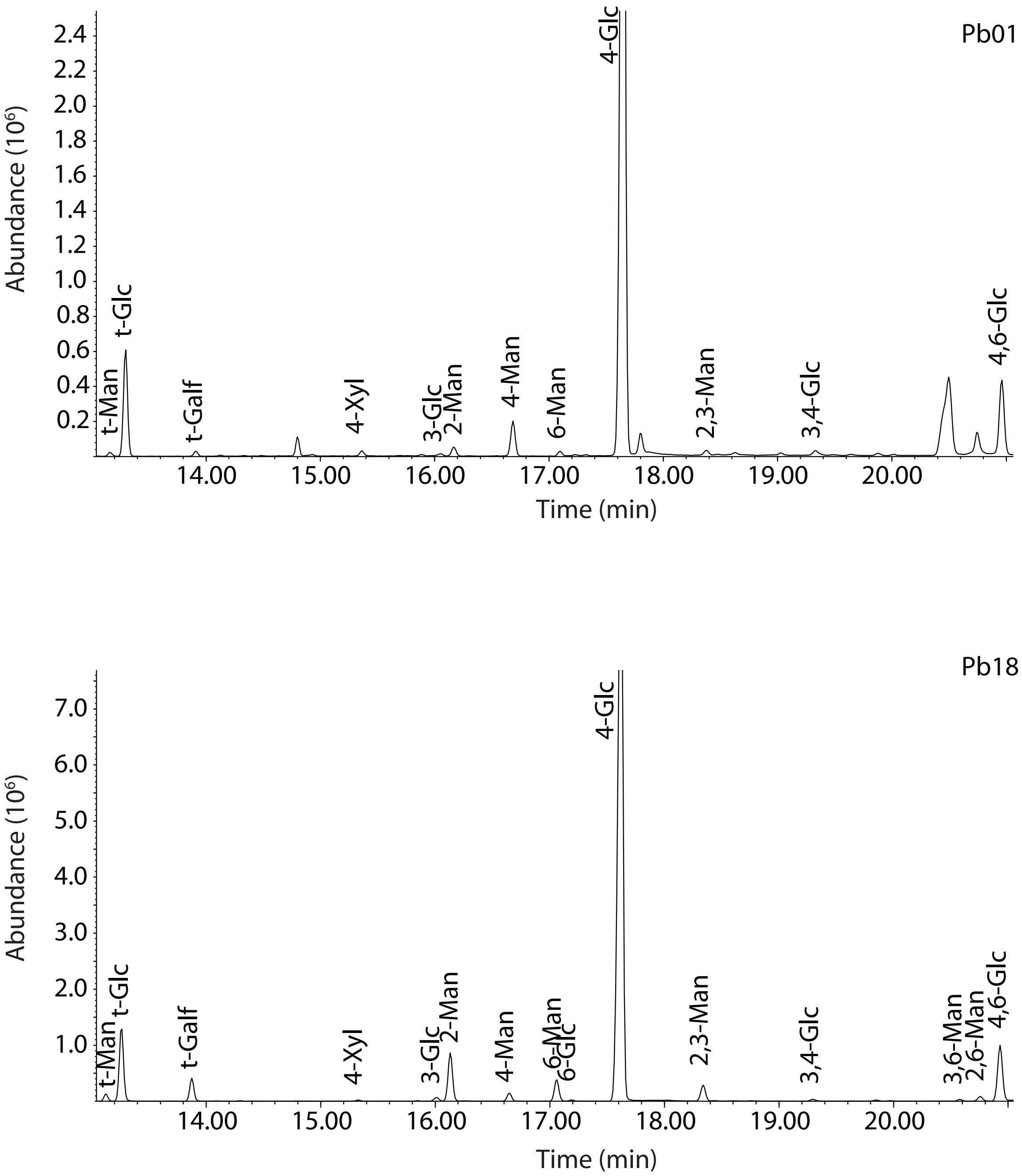

Supplementary Figure 2: GC-MS analysis of glycosyl linkages of PMAAS derived from Pb18 and Pb01 EV total carbohydrate fraction after dialysis. The chromatograms show the relative abundance of monosaccharides found in each isolate sample versus the relative retention time (min) in the fused silica capillary column. The type of linkages and residues identified are indicated in the profiles: t (terminal); Man (mannosyl); Gal (galactopyranosyl); Gal*f* (galactofuranosyl); and Glc (glucosyl).

Supplementary Table 1: NMR chemical shift assignments in ppm for dialyzed Pb18 EV total carbohydrate fraction. Carbon chemical shifts are in italics.

No.	Residue	Chemical shift (ppm)						*NOE*
		1	2	3	4	5	6	
A	α-Glc*p* (t-Glc*p*)	5.36	3.59	3.70	3.43	3.72	3.86/3.77	
		102.5	*74.6*	*75.7*	*72.1*	*75.4*	*63.7*	
B	4-α-Glc*p*	5.36	3.65	3.96	3.64	3.84	3.89/3.77	B-4
		102.5	*74.3*	*76.0*	*80.1*	*74.0*	*63.7*	
C	2-α-Man*p*	5.24	4.11	3.69	3.70	3.77	n.d.	G-2
		103.3	*81.2*	*73.2*	*69.9*	*76.0*	*n.d.*	
D	2,3-α-Man*p*	5.19	4.29	4.00	3.77	n.d.	n.d.	C-2
		103.3	*76.8*	*78.4*	*68.3*	*n.d.*	*n.d.*	
E	β-Gal*f* (t-Gal*f*)	5.19	4.13	4.10	4.07	3.84	3.71/3.67	D-3
		108.0	*83.9*	*79.5*	*85.8*	*73.5*	*65.6*	
F	α-Man*p* (t-Man*p*)	5.09	4.04	n.d.	n.d.	n.d.	n.d.	G-6
		104.1	*73.0*	*n.d.*	*n.d.*	*n.d.*	*n.d.*	
G	2,6-α-Man*p*	5.08	4.02	3.85	3.80	3.77	4.02/3.66	D-2
		101.1	*81.2*	*73.2*	*69.1*	*74.3*	*68.3*	
H	4,6-α-Glc*p*	5.36	3.65	3.96	3.63	3.96	4.02/3.66	B-4
		102.5	*74.3*	*76.0*	*80.1*	*73.0*	*68.3*	

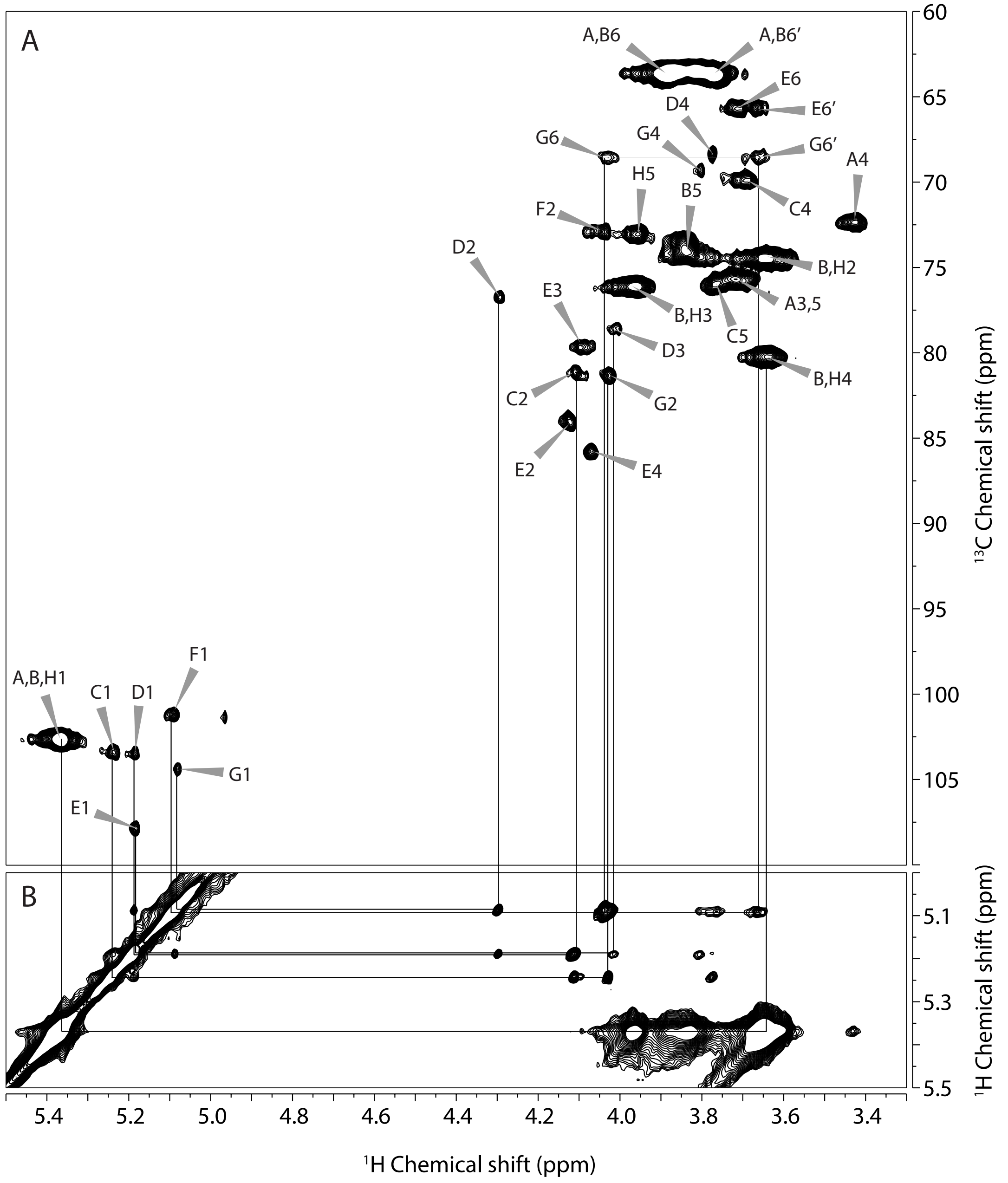

Supplementary Figure 3: Two-dimensional HSQC (A) and partial NOESY (B) NMR spectra of Pb18 EV total carbohydrate fraction after dialysis. The inter-residue NOEs are indicated by solid lines.

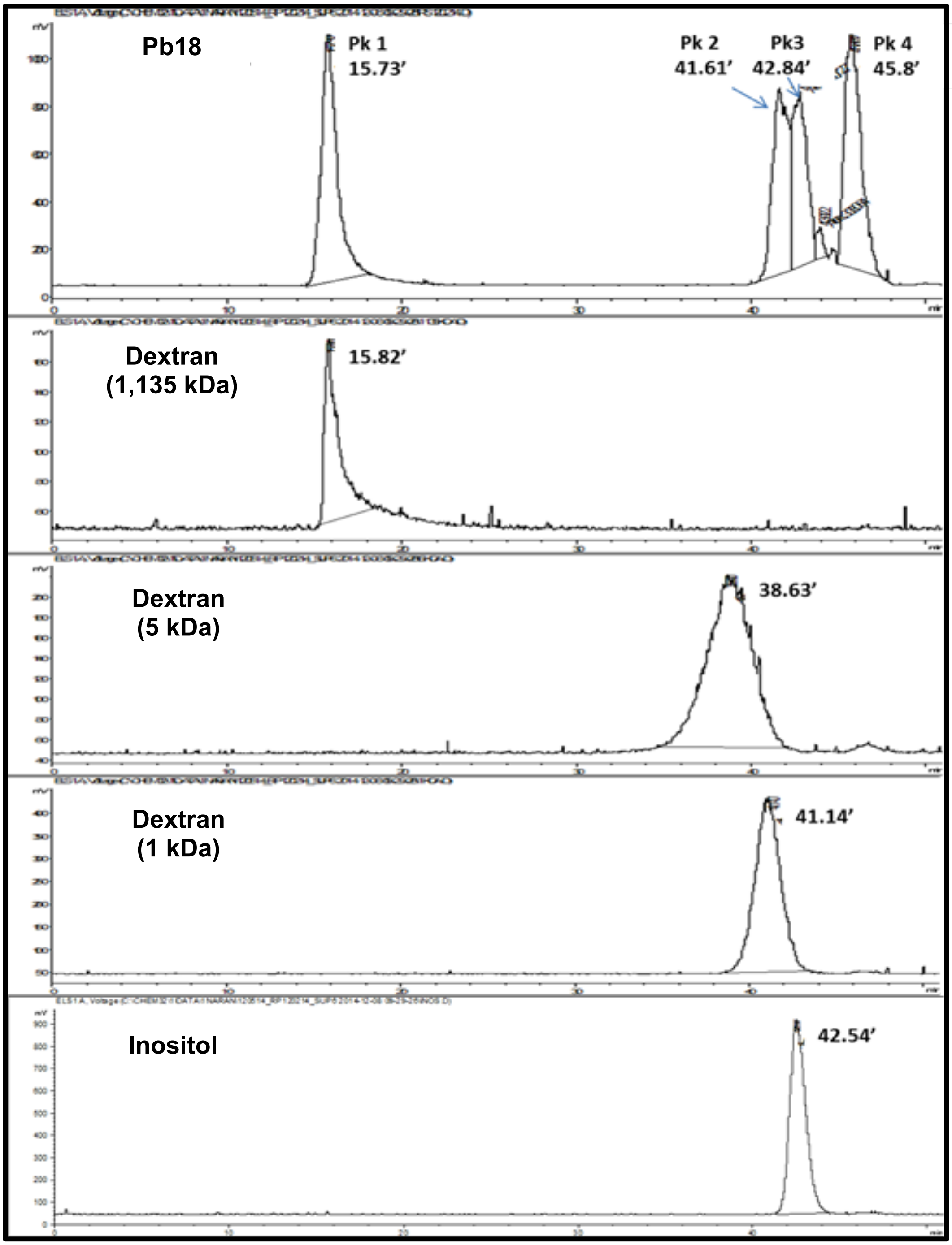

Supplementary Figure 4: Size-exclusion chromatography (SEC) of Pb18 total carbohydrate-enriched fraction. Dextrans with molecular weight (MW) of 1,135, 5, 1 kDa and Inositol were used for calibration of the column. The SEC profile of the sample shows 4 distinct peaks, one over 1,200 kDa, compatible with α-(1,4)-glucan polysaccharide; the second and third are oligosaccharides of molecular mass around 1,000 Da. The peak around 180 Da is likely to correspond to salts that have not been removed during sample fractionation.

Supplementary Table 2: Lectins printed, their binding specificities, their simple print sugars (1 mM) and the supplying company.

Abbreviation	Source	Species	Common name	General binding specificity*	Print sugar	Supplier
AIA, Jacalin	Plant	*Artocarpus integrifolia*	Jack fruit lectin	Gal, Gal-β-(1,3)-GalNAc (sialylation independent)	Gal	EY Labs
RPbAI	Plant	*Robinia pseudoacacia*	Black locust lectin	Gal	Gal	EY Labs
PA-I	Bacteria	*Pseudomonas aeruginosa*	Pseudomonas lectin	Gal, Gal derivatives	Gal	Sigma Aldrich
SNA-II	Plant	*Sambucus nigra*	Sambucus lectin-II	Gal/GalNAc	Gal	EY Labs
SJA	Plant	*Sophora japonica*	Pagoda tree lectin	β-GalNAc	Gal	EY Labs
DBA	Plant	*Dolichos biflorus*	Horse gram lectin	GalNAc	Gal	EY Labs
GHA	Plant	*Glechoma hederacea*	Ground ivy lectin	GalNAc	Gal	EY Labs
SBA	Plant	*Glycine max*	Soy bean lectin	GalNAc	Gal	EY Labs
VVA-B4	Plant	*Vicia villosa*	Hairy vetch lectin	GalNAc	Gal	EY Labs
BPA	Plant	*Bauhinia purpurea*	Camels foot tree lectin	GalNAc/Gal	Gal	EY Labs
WFA	Plant	*Wisteria floribunda*	Japanese wisteria lectin	GalNAc/sulfated GalNAc	Gal	EY Labs
HPA	Animal	*Helix pomatia*	Edible snail lectin	α-GalNAc	Gal	EY Labs
GSL-I-A4	Plant	*Griffonia simplicifolia*	Griffonia isolectin I A4	GalNac	Gal	EY Labs
ACA	Plant	*Amaranthus caudatus*	Amaranthin	Sialylated/Gal-β-(1,3)-GalNAc	Lac	Vector Labs
ABL	Fungus	*Agaricus bisporus*	Edible mushroom lectin	Gal-β(1,3)-GalNAc, GlcNAc	Lac	EY Labs
PNA	Plant	*Arachis hypogaea*	Peanut lectin	Gal-β-(1,3)-GalNAc	Lac	EY Labs
GSL-II	Plant	*Griffonia simplicifolia*	Griffonia lectin-II	GlcNAc	GlcNAc	EY Labs
sWGA	Plant	*Triticum vulgaris*	Succinyl WGA	GlcNAc	GlcNAc	EY Labs
DSA	Plant	*Datura stramonium*	Jimson weed lectin	GlcNAc	GlcNAc	EY Labs
STA	Plant	*Solanum tuberosum*	Potato lectin	GlcNAc oligomers	GlcNAc	EY Labs
LEL	Plant	*Lycopersicum eculentum*	Tomato lectin	GlcNAc-β-(1,4)-GlcNAc	GlcNAc	EY Labs
Calsepa	Plant	*Calystegia sepium*	Bindweed lectin	Man/Maltose	Man	EY Labs
NPA	Plant	*Narcissus pseudonarcissus*	Daffodil lectin	α-(1,6)-Man	Man	EY Labs
GNA	Plant	*Galanthus nivalis*	Snowdrop lectin	Man-α-(1,3)-	Man	EY Labs
HHA	Plant	*Hippeastrum hybrid*	Amaryllis agglutinin	Man-α-(1,3)-Man-α-(1,6)-	Man	EY Labs
ConA	Plant	*Canavalia ensiformis*	Jack bean lectin	Man, Glc, GlcNAc	Man	EY Labs
Lch-B	Plant	*Lens culinaris*	Lentil isolectin B	Man, core fucosylated, agalactosylated biantennary *N*-glycans	Man	EY Labs
Lch-A	Plant	*Lens culinaris*	Lentil isolectin A	Man/Glc	Man	EY Labs

PSA	Plant	*Pisum sativum*	Pea lectin	Man, core fucosylated trimannosyl *N*-glycans	Man	EY Labs
WGA	Plant	*Triticum vulgaris*	Wheat germ agglutinin	NeuAc/GlcNAc	GlcNAc	EY Labs
MAA	Plant	*Maackia amurensis*	Maackia agglutinin	Sialic acid-α-(2,3)-linked	Lac	EY Labs
SNA-I	Plant	*Sambucus nigra*	Sambucus lectin-I	Sialic acid-α-(2,6)-linked	Lac	EY Labs
CCA	Animal	*Cancer antennarius*	California crab lectin	*O*-acetyl sialic acids	Lac	EY Labs
PHA-L	Plant	*Phaseolus vulgaris*	Kidney bean leukoagglutinin	Tri- and tetraantennary β-Gal/Gal-β-(1,4)-GlcNAc	Lac	EY Labs
PCA	Plant	*Phaseolus coccineus*	Scarlet runner bean lectin	GlcNAc in complex oligosaccharides	Lac	Sigma Aldrich
PHA-E	Plant	*Phaseolus vulgaris*	Kidney bean erythroagglutinin	Biantennary with bisecting GlcNAc,β-Gal/Gal-β-(1,4)-GlcNAc	Lac	EY Labs
RCA-I/120	Plant	*Ricinus communis*	Castor bean lectin I	Gal-β-(1,4)-GlcNAc	Gal	Vector Labs
CPA	Plant	*Cicer arietinum*	Chickpea lectin	Complex oligosaccharides	Lac	EY Labs
CAA	Plant	*Caragana arborescens*	Pea tree lectin	Gal-β-(1,4)-GlcNAc	Lac	EY Labs
ECA	Plant	*Erythrina cristagalli*	Cocks comb/coral tree lectin	Gal-β-(1,4)-GlcNAc oligomers	Lac	EY Labs
AAL	Fungi	*Aleuria aurantia*	Orange peel fungus lectin	Fuc-α-(1,6)- and Fuc-α-(1,3)-linked	Fuc	Vector Labs
LTA	Plant	*Lotus tetragonolobus*	Lotus lectin	Fuc-α-(1,3)-, Fuc-α-(1→6)- and Fuc-α-(1→2)-linked	Fuc	EY Labs
UEA-I	Plant	*Ulex europaeus*	Gorse lectin-I	Fuc-α-(1,2)-linked	Fuc	EY Labs
EEA	Plant	*Euonymous europaeus*	Spindle tree lectin	Terminal α-linked Gal	t-Gal	EY Labs
GSL-I-B4	Plant	*Griffonia simplicifolia*	Griffonia lectin-I	Terminal α-linked Gal	t-Gal	EY Labs
MPA	Plant	*Maclura pomifera*	Osage orange lectin	Terminal α-linked Gal	t-Gal	EY Labs
VRA	Plant	*Vigna radiata*	Mung bean lectin	Terminal α-linked Gal	t-Gal	EY Labs
MOA	Fungus	*Marasmius oreades*	Fairy ring mushroom lectin	Terminal α-linked Gal	t-Gal	EY Labs

* Reported recognition based on literature consensus or experimental evidence generated within our laboratory.

Supplementary Table S3: Glycoproteins and neoglyconjugates printed, their abbreviations, supply company and structures. All glycoproteins and neoglycoconjugates were printed at 1 mg/mL except fibrinogen and ovomucoid which were printed at 0.5 mg/mL.

**Abbreviation**	**Neoglycoconjugate**	**Source**	**Structure**
Fetuin	Fetuin	Sigma	
ASF	Asialofetuin	Sigma	
PBS			
Ov	Ovalbumin	Sigma	
RB	RNAse B	Sigma	
Xferrin	Transferrin	Sigma	
4APHSA	4AP-HSA	In house	
a-C	a-Crystallin from bovine lens	Sigma	
M3BSA	Mana1,3(Mana1,6)Man-BSA	Dextra	Man-a-(1,3)-[Man-a-(1,6)-]Man-BSA
GlcNAcBSA	GlcNAc-BSA	Dextra	GlcNAc-sp14-NH2(Lys)-BSA
LacNAcBSA	LacNAc-BSA	Dextra	Gal-b-(1,4)-GlcNAc-sp3-BSA
3SLNBSA	3'SialylLacNAc-BSA	Dextra	
3SLacHSA	3´-Sialyllactose-APD-HSA	IsoSep	Neu5Ac-a-(2,3)-Gal-b-(1,4)(Glc)-APD-HSA
6SLacHSA	6´-Sialyllactose-APD-HSA	IsoSep	Neu5Ac-a-(2,6)-Gal-b-(1,4)(Glc)-APD-HSA
2FLBSA	2'Fucosyllactose-BSA	Dextra	Fuc-a-(1,2)-Gal-b-(1,4)(Glc)-sp3-BSA
3SFLBSA	3'Sialyl-3-fucosyllactose-BSA	Dextra	Neu5Ac-a-(2,3)-Gal-b-(1,4)-[Fuc-a-(1,3)-](Glc)-sp3-BSA
H2BSA	H Type II-APE-BSA	IsoSep	Fuc-a-(1,2)-Gal-b-(1,4)-GlcNAc-b-APE-BSA
BGABSA	Blood Group A-BSA	Dextra	GalNAc-a-(1,3)-[Fuc-a-(1,2)-]Gal-BSA
BGBHSA	Blood Group B-HSA	Dextra	Gal-a-(1,3)-[Fuc-a-(1,2)-]Gal-BSA
GGGNHSA	Gala1,3Galb1,4GlcNAc-HSA	Dextra	Gal-a-(1,3)-Gal-b-(1,4)-GlcNAc-HSA
Ga3GBSA	Gala1,3Gal-BSA	Dextra	Gal-a-(1,3)-Gal-sp3-BSA
Gb4GBSA	Galb1,4GalBSA	Dextra	Gal-b-(1,4)-Gal-sp3-BSA
Ga2GBSA	Gala1,2GalBSA	Dextra	Gal-a-(1,2)-Gal-sp3-BSA
4APBSA	4AP-BSA	In house	
LNFPIBSA	Lacto-*N*-fucopentaose I-BSA	Dextra	Fuc-a-(1,2)-Gal-b-(1,3)-GlcNAc-b-(1,3)-Gal-b-(1,4)(Glc)-BSA
LNFPIIBSA	Lacto-*N*-fucopentaose II-BSA	Dextra	Fuc-a-(1,3)-Gal-b-(1,3)-GlcNAc-b-(1,3)-Gal-b-(1,4)(Glc)-BSA
LNFPIIIBSA	Lacto-*N*-fucopentaose III-BSA	Dextra	Gal-b-(1,4)-[Fuc-a-(1,3)-]GlcNAc-b-(1,3)-Gal-b-(1,4)(Glc)-BSA
LNDHIBSA	Lacto-*N*-difucohexaose I-BSA	Dextra	Fuc-a-(1,2)-Gal-b-(1,3)-[Fuc-a-(1-4)-]GlcNAc-b-(1,3)-Gal-b-(1,4)(Glc)-sp3-BSA
LebBSA	LNDI-BSA/ Lewis b-BSA	IsoSep	Fuc-a-(1,2)-Gal-b-(1,3)-[Fuc-a-(1,4-)-]GlcNAc-b-(1,3)-Gal-b-(1,4)(Glc)-APD-BSA
LexBSA	Lewis x-BSA	Dextra	Gal-b-(1,4)-[Fuc-α-(1,3)-]GlcNAc-BSA

DiLexBSA	Di-Lex-APE-BSA	IsoSep	Gal-b-(1,4)-[Fuc-a-(1,3)-]GlcNAc-b-(1,3)-Gal-b-(1,4)-[Fuc-a-(1,3)-]GlcNAc-b-APE-BSA
DiLexHSA	Di-Lewis x-APE-HSA	IsoSep	Gal-b-(1,4)-[Fuc-a-(1,3)-]GlcNAc-b-(1,3)-Gal-b-(1,4)-[Fuc-a-(1,3)-]GlcNAc-b-APE-HSA
3LexHSA	Tri-Lex-APE-HSA	IsoSep	Gal-b-(1,4)-[Fuc-a-(1,3)-]GlcNAc-b-(1,3)-Gal-b-(1,4)-[Fuc-a-(1,3-]GlcNAc-b-(1,3)-Gal-b-(1,4)-[Fuc-a-(1,3)-]GlcNAc-b-APE-HSA
3SLexBSA3	3'Sialyl Lewis x-BSA	Dextra	Neu5Ac-a-(2,3)-Gal-b-(1,4)-[Fuc-a-(1,3)-]GlcNAc-sp3-BSA
SLexBSA14	3'Sialyl Lewis x-BSA	Dextra	Neu5Ac-a-(2,3)-Gal-b-(1,4)-[Fuc-a-(1,3)-]GlcNAc-sp14-BSA
6SuLexBSA	6-Sulfo Lewis x-BSA	Dextra	Gal6SO_4-b-(1,4)-[Fuc-a-(1,3)-]GlcNAc-sp3-BSA
6SuLeaBSA	6-Sulfo Lewis a-BSA	Dextra	Gal6SO_4-b-(1,3)-[Fuc-a-(1,4)-]GlcNAc-sp3-BSA
3SuLeaBSA	3-Sulfo Lewis a-BSA	Dextra	Gal3SO_4-b-(1,3)-[Fuc-a-(1,4)-]GlcNAc-BSA
3SuLexBSA	3-Sulfo Lewis x-BSA	Dextra	Gal3SO_4-b-(1,4)-[Fuc-a-(1,3)-]GlcNAc-BSA
DFPLNHHSA	Difucosyl-para-lacto-N-hexaose-APD-HSA (Lea/Lex)	IsoSep	Gal-b-(1,3)-[Fuc-a-(1,4)-]GlcNAc-b-(1,3)-Gal-b-(1,4)-[Fuc-a-(1,3)-]GlcNAc-b-(1,3)-Gal-b-(1,4)(Glc)-APD-HSA
LeaBSA	Lewis a-BSA	Dextra	Gal-b-(1,3)-[Fuc-a-(1,4)-]GlcNAc-sp3-BSA
LeyHSA	Lewis y-tetrasaccharide-APE-HSA	IsoSep	Fuc-a-(1,2)-Gal-b-(1,4)-[Fuc-a-(1,3)-]GlcNAc-b-APE-HSA
3FLeyHSA	Tri-fucosyl-Ley-heptasaccharide-APE-HSA	IsoSep	Fuc-a-(1,2)-Gal-b-(1,4)-[Fuc-a-(1,3)-]GlcNAc-b-(1,3)-Gal-b-(1,4)-[Fuc-a-(1,3)-]GlcNAc-b-APE-HSA
LNnTHSA	Lacto-N-neotetraose-APD-HSA	IsoSep	Gal-b-(1,4)-GlcNAc-b-(1,3)-Gal-b-(1,4)(Glc)-APD-HSA
LNTHSA	Lacto-N-tetraose-APD-HSA	IsoSep	Gal-b-(1,3)-GlcNAc-b-(1,3)-Gal-b-(1,4)(Glc)-APD-HSA
SLNFVHSA	Sialyl-LNF V-APD-HSA	IsoSep	Fuc-a-(1,2)-Gal-b-(1,3)-[NeuAc-a-(2,6)-]GlcNAc-b-Gal-b-(1,4)(Glc)-APD-HSA
MMLNnHHSA	Monofucosyl, monosialyllacto-N-neohexaose-APD-HSA	IsoSep	Neu5Ac-a-(2,3)-Gal-b-(1,4)-GlcNAc-b-(1,3)-[Gal-b-(1,4)-[Fuc-a-(1,3)-]GlcNAc-b-(1,6)-]Gal-b-(1,4)(Glc)-APD-HSA
SLNnTHSA	Sialyl-LNnT-penta-APD-HSA	IsoSep	Neu5Ac-a-(2,3)-Gal-b-(1,4)-GlcNAc-b-(1,3)-Gal-b-(1,4)(Glc)-APD-HSA
GM1HSA	GM1-pentasaccharide-APD-HSA	IsoSep	Gal-b-(1,3)-GalNAc-b-(1,4)-[Neu5Ac-a-(2,3)-]Gal-b-(1,4)(Glc)-APD-HSA
aGM1HSA	Asialo-GM1-tetrasaccharide-APD-HSA	IsoSep	Gal-b-(1,3)-GalNAc-b-(1,4)-Gal-b-(1,4)(Glc)-APD-HSA
GlobNTHSA	Globo-N-tetraose-APD-HSA	IsoSep	GalNAc-b-(1,3)-Gal-a-(1,4)-Gal-b-(1,4)(Glc)-APD-HSA
GlobTHSA	Globotriose-APD-HSA	IsoSep	Gal-a-(1,4)-Gal-b-(1,4)-Glc-b-APE-HSA
Inv	Invertase	Sigma	
Fibrin	Fibrinogen		
A1AT	alpha-1-antitrypsin	Sigma	
4APHSA	4AP-HSA	In house	
Cerulo	Ceruloplasmin		

AGP	alpha-1-acid glycoprotein	Sigma	
LacNAcaBSA	LacNAc-a-4AP-BSA	In house	
LacNAcb4APBSA	LacNAc-b-4AP-BSA	In house	
H2HSA	H-Type 2-APE-HSA	IsoSep	Fuc-a-(1,2)-Gal-b-(1,4)-GlcNAc-b-APE-HSA
Ovomuc	Ovomucoid	Sigma	
RhaBSA	L-Rhamnose-sp14-BSA	Dextra	
GalaPITCBSA	Gal-a-PITC-BSA	In house	
XManaBSA	Man-a-ITC-BSA	In house	
XLacbBSA	Lac-b-4AP-BSA	In house	
XManbBSA	Man-b-4AP-BSA	In house	
XGalbBSA	Gal-b-ITC-BSA	In house	
XylbBSA	Xyl-b-4AP-BSA	In house	
XylaBSA	Xyl-a-4AP-BSA	In house	
XGlcbBSA	Glc-b-4AP-BSA	In house	
FucaBSA	Fuc-a-4AP-BSA	In house	
FucbBSA	Fuc-b-4AP-BSA	In house	
GlcbITCBSA	Glc-b-ITC-BSA	In house	
Galb4APBSA	Gal-b-4AP-BSA	In house	
Neu5GcBSA	Neu5Gc-4AP-BSA	In house	
D-GlobTHSA	Globotriose-HSA	Dextra	Gal-a-(1,4)-Gal-b-(1,4)(Glc)-sp3-BSA

Supplementary Table S4: Mammalian lectins printed, their abbreviations and their simple print sugars (1 mM). All lectins were from R&D Biosystems and were printed at 0.5 mg/mL.

Abbreviation	Lectin	Print sugar
hESel_5_G	Human E-selectin	Glc
hESel_5_S	Human E-selectin	Suc
hLSel_5_L	Human L-selectin	Lac
hLSel_5_S	Human L-selectin	Suc
hPSel_5_L	Human P-selectin	Lac
hPSel_5_S	Human P-selectin	Suc
hSig-1_5_S	Human siglec-1	Suc
hSig-1_5_SA	Human siglec-1	Neu5Ac
hSig-2_5_S	Human siglec-2	Suc
hSig-2_5_SA	Human siglec-2	Neu5Ac
rMAG_5_S	Rat MAG	Suc
rMAG_5_SA	Rat MAG	Neu5Ac
hSig-10_5_S	Human siglec-10	Suc
hSig-10_5_SA	Human siglec-10	Neu5Ac
DCSIGN_5_M	DC-SIGN	Man
DCSIGN_5_S	DC-SIGN	Suc
DCSIGNR_5_M	DC-SIGNR	Man
DCSIGNR_5_S	DC-SIGNR	Suc
Dec1_5_G	Dectin-1	Glc
Dec1_5_S	Dectin-1	Suc
Dec2_5_M	Dectin-2	Man
Dec2_5_S	Dectin-2	Suc
Lsec_5_M	LSECtin	Man
Lsec_5_S	LSECtin	Suc
MMR_5_M	Mannose macrophage receptor	Man
MMR_5_S	Mannose macrophage receptor	Suc
Fic-1_SA	Human ficolin-1	Neu5Ac
Fic-1_S	Human ficolin-1	Suc
Fic-2_GN	Human ficolin-2	GlcNAc
Fic-2_S	Human ficolin-2	Suc
Fic-3_M	Human ficolin-3	Man
Fic-3_S	Human ficolin-3	Suc
